# Individual Differences in Brain Structure and Resting Brain Function Underlie Cognitive Styles: Evidence from the Embedded Figures Test

**DOI:** 10.1371/journal.pone.0078089

**Published:** 2013-12-13

**Authors:** Xin Hao, Kangcheng Wang, Wenfu Li, Wenjing Yang, Dongtao Wei, Jiang Qiu, Qinglin Zhang

**Affiliations:** 1 Key laboratory of cognition and personality (SWU), Ministry of Education, Chongqing, China; 2 Department of Psychology, Southwest University, Chongqing, China; National Research & Technology Council, Argentina

## Abstract

Cognitive styles can be characterized as individual differences in the way people perceive, think, solve problems, learn, and relate to others. Field dependence/independence (FDI) is an important and widely studied dimension of cognitive styles. Although functional imaging studies have investigated the brain activation of FDI cognitive styles, the combined structural and functional correlates with individual differences in a large sample have never been investigated. In the present study, we investigated the neural correlates of individual differences in FDI cognitive styles by analyzing the correlations between Embedded Figures Test (EFT) score and structural neuroimaging data [regional gray matter volume (rGMV) was assessed using voxel-based morphometry (VBM)] / functional neuroimaging data [resting-brain functions were measured by amplitude of low-frequency fluctuation (ALFF)] throughout the whole brain. Results showed that the increased rGMV in the left inferior parietal lobule (IPL) was associated with the EFT score, which might be the structural basis of effective local processing. Additionally, a significant positive correlation between ALFF and EFT score was found in the fronto-parietal network, including the left inferior parietal lobule (IPL) and the medial prefrontal cortex (mPFC). We speculated that the left IPL might be associated with superior feature identification, and mPFC might be related to cognitive inhibition of global processing bias. These results suggested that the underlying neuroanatomical and functional bases were linked to the individual differences in FDI cognitive styles and emphasized the important contribution of superior local processing ability and cognitive inhibition to field-independent style.

## Introduction

Cognitive styles refer to individual differences in the way people perceive, think, learn, solve problems, and relate to others [1-3]. Many cognitive style dimensions have been studied in the literature, however, field dependence/independence (FDI) is the most widely studied dimension measured by Embedded Figures Test (EFT) [4,5]. The EFT requires subjects to locate the simple shape that embedded in a complex figure. Based on the EFT score, Witkin et al. [1] identified the field-dependent (FD) and field-independent (FI) visual perceptual styles. FD individuals exhibit more dependency on the surrounding field and cannot easily perceive the embedded part. One the other extreme, FI individuals tend to be less influenced by the information from the visual fields and can perform better in the test [1,5]. The EFT score forms a continuous distribution, and reflects a varying degrees towards one kind of perception tendency mode or the other [1,2]. A person’s tendency to perceive was found to be significantly related to their cognitive functioning, personality characteristic, and their social behavior [5]. At the present time, FDI cognitive styles have been used as an excellent predictor of an individual’s success in a particular situation, particularly in terms of academic achievement, individual and organizational behaviors in numerous applied fields [6,7]. FDI cognitive styles are widely studied in cognitive and educational fields, but the neural underpinnings of FDI cognitive styles have been little investigated.

To date, a limited number of studies employed neuropsychological measures to investigate FDI cognitive styles that mainly focused on visuospatial processing bias and clinical individual differences [8–15]. Early EEG studies showed that FI subjects exhibited smaller between-hemisphere coherence and more hemispheric specialization [8,9]. The successful performance in the EFT reflects individual variations in detecting local features under the circumstance that global perception still dominates [10]. Manjaly et al. [11]used a straightforward shape recognition task as control condition, and found significant activations in the left inferior and superior parietal cortex and left inferior frontal gyrus specific in the EFT. Furthermore, Lee et al. [12]adopted a match task as control condition and suggested that posterior cortical in the left hemisphere was related to the perception of local elements and the medial frontal involved in the suppression of global perceptual processing bias in EFT. Recently, Walter and Dassonville [14] found that FI individuals typically recruit a strongly bilateral frontoparietal network when performing the EFT. Many clinical studies found that children with high-functioning autism exhibited superior performance on the EFT [16-19]. Autism is possibly characterized by a cognitive style biased towards local rather than global information processing [20,21]. Although FI doesn’t equate with autism, neuroimaging studies on autism may help us to understand more specific regional functions. Damarla et al. [15] observed that more activation occurred in left dorsolateral prefrontal and inferior parietal lobe in normal control subjects and more activation occurred in visuospatial areas in autism group. They suggested normal subjects had more functional integration of higher-order executive regions with visuospatial regions, while autism relied more on visuospatial regions to preserve or enhance performance on the EFT. Upon these studies, we supposed that individual differences in FDI cognitive styles might be related to the differential use of medial frontal region for suppression of the irrelevant background information or global processing bias and posterior visual-spatial regions (inferior/superior parietal regions) for local visual processing.

Despite previous task-related neuroimaging studies on FDI cognitive styles described above, these studies depended heavily on inconsistent methodologies and limited by a small sample size, which lead difficult to reconcile the inconsistencies among them [22]. Moreover, previous studies have stated that people are quite stable in their preferred perception mode [23,24]. On the other hand, the superiority on the EFT is the cognitive profile characteristic of autism individual, which has strong heritability [25,26]. Baron-Cohen and Hammer [27] reported that the parents of children with autism or Asperger’s syndrome were also faster on the EFT relative to the matched control parents. Considering its stability, examining structure correlates of FDI cognitive styles would eliminate task-related differences and become especially useful for investigating the anatomical correlates of individual differences. 

On a similar note, resting state shows strong activation of several brain areas without an external task [28]. Spontaneous cortical activity can serve as a predictor of individual differences in several cognitive domains, such as perception, problem solving, and memory [29–31]. The spontaneous fluctuations in the blood oxygen level dependent (BLOD) signal of fMRI are not random noise but physiologically meaningful and low-frequency fluctuations (LFFs; 0.01 Hz to 0.08 Hz) reveal spontaneous neuronal activity [32]. Moreover, regional amplitude of low frequency fluctuation (ALFF) was an index for measuring regional spontaneous neuronal activity in the resting-state fMRI [33–35]. 

Functional imaging studies have shown that the increases in gray matter (GM) are associated with increased or decreased brain activity [36–38]. The structure and function may function differently and change independently [39]. For these reasons, the combination of structural imaging of regional gray matter volumes and ALFF of resting state would provide complementary information and advance our understanding of the FDI cognitive styles. However, the neural substrate of individual differences in FDI cognitive styles that employed the combined methods has never been investigated yet. 

In this study, fMRI was performed to examine both structural and resting-state functional brain alterations in FDI cognitive styles measured by EFT. Structural differences related to FDI was first conducted to be examined by standard voxel-based morphometry (VBM) [40]. And the ALFFs [34,41]of resting-state fMRI were used to reflect regional properties of the brain’s intrinsic neural activity. Furthermore, based on previous neuroimaging studies on FDI cognitive styles, we predicted that FDI cognitive styles would be associated with visuospatial processing and high-order suppression, subserved mainly by significant structural and functional alterations in posterior visual-spatial regions and medial frontal region respectively. The combination of structural and resting-state functional data may improve our understanding of the neural correlates of individual differences in FDI cognitive styles. 

## Methods

### Ethics statement

The study was approved by Southwest University Brain Imaging Center Institutional Review Board. In accordance with the Declaration of Helsinki (1991), written informed consents were obtained from all participants. In addition, all of the participants were remunerated for their participation.

### Participants

A total of 286 right-handed, healthy volunteers (140 females and 146 males; mean age = 20.01 years, SD = 1.33, aged 18-26 years) participated in the study as part of our ongoing project to examine the association among brain imaging, creativity and mental health. For VBM analyses, all 286 subjects were included in the study. For ALFF analyses, 25 subjects were excluded due to excessive head motions, resulting in 261 subjects (132 females and 129 males; mean age = 20.03 years, SD=1.36, aged 18-26 years). Data derived from the subjects in this study are to be used in other studies irrelevant to the theme of this study. Our project gathered psychological behavioral data and imaging data for every subject. Behavioral measures consist of questionnaires for creativity, personality, intelligence, and mental health and experimental tasks for working memory, attention, reponse inhibition, emotion Stroop task. MRI scans included resting state imaging, T1-weighted image and diffusion tensor imaging. All participants were university students from the local community of Southwest University. They were recruited using adverts on bulletin board at BBS of Southwest University (http://qcjy.swu.edu.cn/bbs/) or by introducing this study and our laboratory’s previous experiments by person in charge in every college to our subjects. No participant had a history of neurological or psychiatric illness. 

### Embedded figures test

EFT is a timed paper-and-pencil performance test adapted from the individual-administered Embedded Figures Test [42]. The present study employed a Chinese version of the EFT revised by the College of Psychology in Beijing Normal University [43]. The revised EFT adopts the majority of the original EFT items, with a few complex figures slightly modified. It comprises three sections: the first/practice section (9 figures); section B (10 figures); and section C (10 figures). The task is to locate and trace the simple figures in the context of the complex figures, as quickly as possible within three 5-min sections (the practice section, section B, section C). The total number of correct answers on the second and third sections (ranged between 0 and 20) were considered as the EFT score.

These modifications to the original EFT were based on much polit testing in four groups (adults, senior-high-school students, junior-high-school students, primary-school students). Validity had been tested by Pearson’s correlation coefficients between EFT scales and the rod-and-frame test (RFT) [*r* = 0.49, *p* < 0.05]. The test reliability was calculated by Pearson’s correlation coefficients between section B and section C of the revised EFT [*r* = 0.90, *p* < 0.05]. The difficulty distribution of the revised EFT is 0.97-0.21; the discrimination distribution of it is 0.17-0.94.

### Assessment of general intelligence

The Raven’s Progressive Matrices test, which is often regarded as a good marker of the general factor of fluid intelligence [44]. In this study, the Chinese version of the combined Raven’s Progressive Matrices test (CRT) was used for fluid intelligence [45-47]. The CRT is composed of the Colored Progressive Matrices (A, B, and AB sets) and the last three parts of the Standard Progressive Matrices (C, D, and E sets). Each set comprises five items increasingly difficulty. The number of the correct answers given in 40 min was used as the CRT score. The CRT scale has high internal consistency (Cronbach’s α = 0.93) and a good validity (*r* = 0.56) with another popular general intelligence scale, namely, Wechsler Intelligence Scale [45–47].

### MRI Data Acquisition

All of the MR images were acquired on a 3.0 T Siemens Trio MRI scanner (Siemens Medical, Erlangen, Germany) at the Brain Imaging Research Central in Southwest University. For each subject two sets of MR images were acquired in this study. First, subjects completed a resting-state functional scan, during which time they were instructed to close eyes, not to move, think particularly or fall asleep. Each subject reported not having fallen asleep using a simple questionnaire after scanning. BOLD images were obtained using Echo Planar Imaging (EPI) sequence with following parameters: slices = 28; repetition time (TR)/echo time (TE) = 2000/40 ms; flip angle = 90°; FOV = 256 mm × 256 mm; voxel size = 4 ms × 4 ms × 4 ms; thickness/slice gap = 4/1 mm; and matrix = 64 × 64. For each subject, total 242 volumes were collected. Second, a high-resolution T1-weighted anatomical images were acquired using a magnetization-prepared rapid gradient echo (MPRAGE) sequence (TR = 1900ms; TE = 2.52 ms; inversion time = 900 ms; flip angle = 9 degrees; resolution matrix = 256 × 256; slices = 176; thickness = 1.0 mm; voxle size = 1 mm × 1 mm × 1 mm).

### VBM analysis

The MR images were processed using the SPM8 (Wellcome Department of Cognitive Neurology, London, UK; www.fil.ion.ucl.ac.uk/spm/) implemented in Matlab 7.8 (MathWorks Inc., Natick, MA, USA). Each MR image was first displayed in SPM8 to screen for artifacts or gross anatomical abnormalities. For better registration, the reorientation of the images was manually set to the anterior commissure. Segmentation of the images into gray matter (GM), white matter (WM) and cerebrospinal fluid (CSF) using the new segmentation in SPM8. Subsequently, we performed Diffeomorphic Anatomical Registration through Exponentiated Lie (DARTEL) algebra in SPM8 for registration, normalization, and modulation [48]. To ensure that regional differences in the absolute amount of GM were conserved, the image intensity of each voxel was modulated by the Jacobian determinants. Then, registered images were transformed to standard Montreal Neurological Institute (MNI) space. Finally, the normalized modulated images (gray matter images) were smoothed with a 10-mm full-width at half maximum (FWHM) Gaussian kernel to increase signal to noise ratio. 

Statistical analyses of GMV data were performed using SPM8. In the whole-brain analyses, we used a multiple linear regression to identify regions where regional GMV was associated with individual differences in EFT score. In the multiple linear regression analyses, the score of EFT was used as the variable of interest. To control for possible confounds variables, age, sex, the CRT score and global volumes of GM were entered as covariates into the regression model. To reduce the risk of false negatives and achieve maximal sensitivity, we applied explicit masking with an population-specific automatic optimal threshold to restrict the search volume within gray matter achieved using the Masking toolbox in SPM8 (http://www0.cs.ucl.ac.uk/staff/g.ridgway/masking/). This automatic mask-creation strategy is based on maximizing the correlation between the original and thresholded images and attempts to find an optimal threshold to binarize an average image [49].. At the whole-brain level, a multiple comparison correction was performed using the voxel-level False Discovery Rate (FDR) approach, at a threshold of *p* <0.05 [50]. 

### ALFF analysis

Functional image preprocessing was performed using Data Processing Assistant for Resting-state fMRI (DPARSF,http://www.restfmri.net/forum/DPARSF;[51]) software. The first ten volumes of the functional images were discarded, because of the instability of the initial MR signals and subjects’ adaptation to the circumstances. The remaining images were preprocessed following these steps: slice timing correction, head motion correction, spatial normalization to the Montreal Neurological Institute (MNI) template and then resampling voxel size of 3 mm× 3 mm×3 mm followed by spatial smoothing with a 8-mm full width at half maximum (FWHM) Gaussian kernel. 25 subjects with an estimated maximum displacement in any direction greater than 2.0 mm or a head rotation greater than 2.0° were discarded to minimize movement artifacts in this study. The time series were transformed to the frequency domain using a fast Fourier transform (FFT). Then the power spectrum obtained was square-rooted and averaged across 0.01–0.08 Hz at each voxel. This averaged square root comprised the ALFF. Finally, the ALFF value of each voxel was standardized by dividing the global mean ALFF value within a brain-mask, and other tissues outside the brain were removed [34,52].

A multiple linear regression was performed to identify regions where regional ALFF was associated with EFT score at the whole-brain level. Age, sex and the CRT score were entered as covariates of no interest into the regression model. The score of EFT was used as the variable of interest. A more lenient correction was adopted for multiple comparisons. Clusters were considered significant at the combined voxel-extent threshold of an uncorrected voxel level of *p* < 0.001 and cluster extent > 49 voxels, which corresponded to a corrected *p* < 0.005. The AlphaSim correction (cluster radius connection: *rmm* = 5; number of Monte Carlo simulations = 1000) was conducted using the AlphaSim program in the REST software (http://www.restfmri.net), which applied Monte Carlo simulation [53] to caculate the probability of false positive detection by considering both the individual voxel probability thresholding and cluster size [51]. 

## Results

### Sample Descriptive

Table 1 lists the characteristics of demographics of the total sample. A total of 286 healthy subjects (140 females and 146 males) were included in the VBM analysis, and 261 subjects (132 females and 129 males) were included in the ALFF analysis. The EFT score for all subjects ranged from 6 to 20. There were no significant differences between sexes on the EFT score of either the VBM or ALFF analysis (see Table 1).

**Table 1:** Demographic and behavioral data.

Items	Total subjects
**VBM analysis**	
Number of subjects	286
Females / males	140 / 146
Age (years)	20.01 ± 1.33 (18-26)
CRT score	65.90 ± 3.45 (49-72)
EFT score	13.66 ± 3.03 (6-20)
**ALFF analysis**	
Number of subjects	261
Females / males	132 / 129
Age (years)	20.03 ± 1.36 (18-26)
CRT score	65.89 ± 3.42 (49-72)
EFT score	13.63 ± 3.07 (6-20)

A total of 286 subjects were included in the VBM analysis. From that sample, 261 subjects were included in the ALFF analysis.

Abbreviations: VBM, voxe-based morphometry; ALFF, amplitude of low-frequency fluctuation; EFT, embedded figures test.

### Correlation between rGMV/ALFF and EFT Score

**Figure 1:**
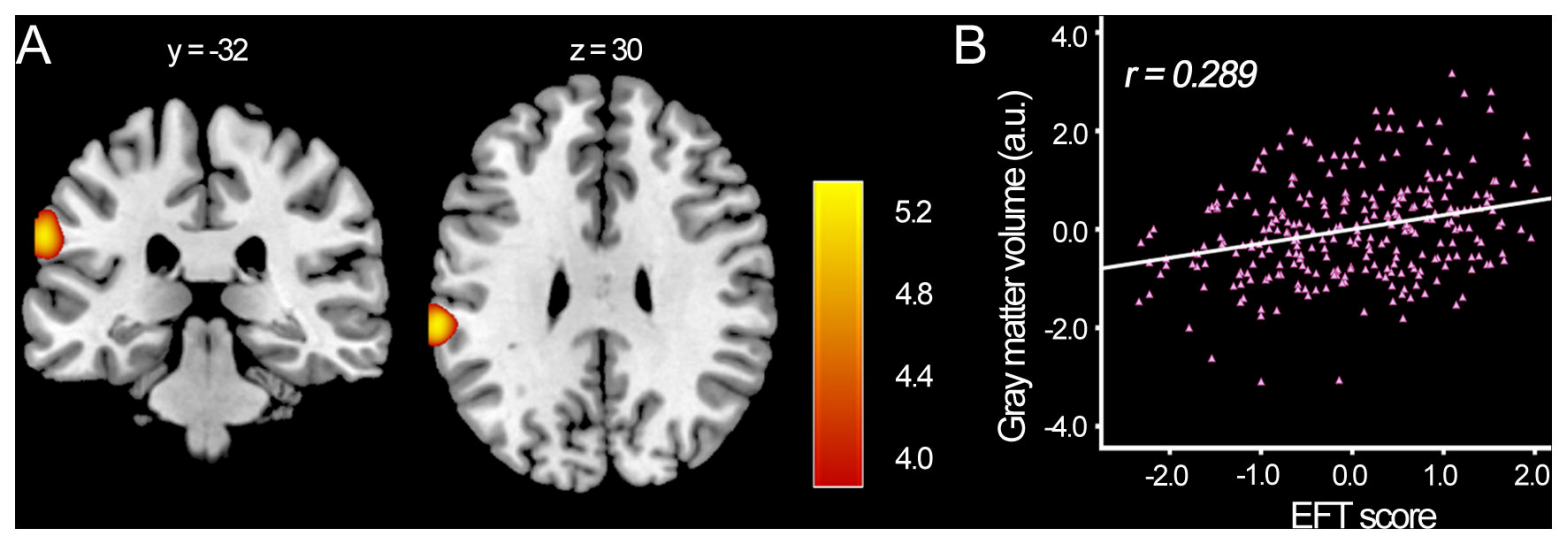
Regions of correlation between rGMV and EFT score (***p* < 0.05, corrected for FDR)**. (A) The left IPL in which variability in rGMV exhibited significant positive correlation with EFT score (n=286) is superimposed on a standard T1-weighted template brain in MNI stereotactic space. (B) A scatterplot between left IPL volume and EFT score adjusted for age, gender, and total gray matter volume is shown for illustration purpose only.

**Figure 2:**
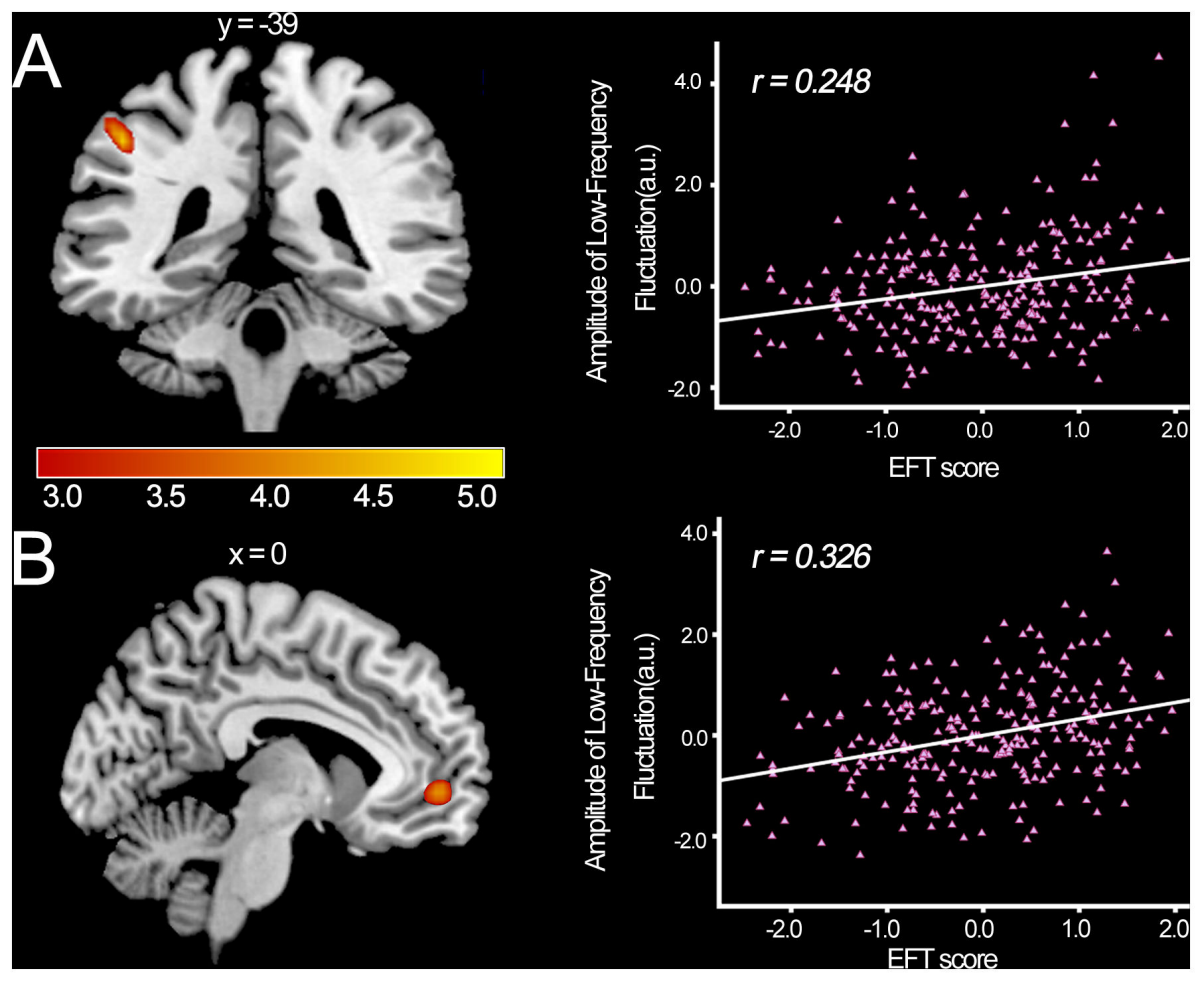
ALFF was positively correlated with individual EFT score. In the left panel, results are shown with p < 0.0005 uncorrected for visualization purposes. (A) Coronal view. Regions of significant correlation are shown in the left IPL; (B) Sagittal view. Regions of significant correlation are shown in the mPFC. The right panel shows corresponding scatterplots of the relationship between the EFT score and ALFF values of the significant cluster in the region of the left panel. The scatterplots adjusted for age, gender, CRT score are shown for illustration purpose only.

After controlling for age, gender, CRT socre and global GM volumes, EFT score was positively correlated with the gray matter volume in a cluster that mainly included areas in the inferior parietal lobule (IPL) [left: r = 0.289, cluster size = 661, t = 5.34, p (corr) = 0.001; see Figure 1, Table 2]. There was no brain area whose GMV was negatively correlated with EFT score. With a more lenient threshold, EFT score was only positively correlated with ALFF values in the IPL [left: r = 0.248, cluster size = 97, *t* = 4.74, *p* (*corr*) = 0.005; see Figure 2, Table 2] and mPFC [ *r* = 0.326, cluster size = 63, *t* = 4.15, *p* (*corr*) = 0.005; see Figure 2, Table 2]. Although the increased GMV in left IPL was found almost near the increased ALFF in the same region, there was no overlapping between the GMV and ALFF results, and the GMV in the regions did not correlate with the ALFF.

**Table 2:** Brain regions with significant positive correlations between rGMV/ALFF values and EFT score.

analysis	Brian regions	BA	Peak coordinates			Cluster size (voxels)	*t*-value
			*x*	*y*	*z*		
VBM	Left IPL	40	-65	-32	30	661	5.34
ALFF	Left IPL	40	-48	-39	45	97	4.74
	mPFC	10	0	48	-6	63	4.15

No regions showed significant negative correlations between rGMV/ALFF and EFT score.

Abbreviations: rGMV, regional gray matter volume; ALFF, amplitude of low-frequency fluctuation; EFT, embedded figures test; BA, Brodmann areas; IPL, inferior parietal lobule; mPFC, medial prefrontal cortex.

## Discussions

To the best of our knowledge, this is the first study combined VBM analysis of structural neuroimaging data and ALFF analysis of resting-state fMRI data to investigate the association between FDI cognitive styles as measured by EFT and rGMV/ALFF in subcortical regions. Our findings showed that increased rGMV in the left IPL was associated with EFT score. Consistent with our expectations, a significant positive correlation between ALFF and EFT score was found in the fronto-parietal network, including left IPL and mPFC. The IPL obtained by VBM and ALFF analyses did not overlap. Furthermore, no negative correlations in neither rGMV nor ALFF were observed. Our results suggested that underlying neuroanatomical and functional bases were linked to the individual differences in FDI cognitive styles. 

Firstly, greater rGMV in the left IPL may contribute to superior local visual processing for more efficient performers (FI individuals). This result corresponded to the activation pattern and findings of other functional visual-spatial imaging studies [11,54-60]. Therefore we considered that left IPL might be the core brain region involved in detail-focused visuospatial task (specific in EFT). EFT assesses visuospatial tendency indicative of spatial orienting or global-local processing [60,61]. Fink et al. [62] found that inferior parietal regions were involved in locating an object in space or making judgments about related object properties. Strong local processing requirements in EFT was well-established left-hemispheric dominance in Manjaly’s study [13] and more activation was observed in left posterior parietal cortex, including the intraparietal sulcus (IPS) and left ventral premotor cortex (posterior inferior parietal lobe). Furthermore, previous studies have demonstrated that individuals with autism exhibit superior ability in terms of spatial abilities and attention-to-detail [27,63]. They found that children with autism or Asperger’s syndrome were more accurate and faster than normal on the EFT [17,19] and these superior characteristics of autism has strong heritability [25,27,64]. Damarla et al. [15] reported autism group more relied on visuospatial areas (bilateral superior extending to inferior parietal and right occipital) areas for the intact or superior performance in EFT. These studies suggested that performance on the EFT reflected the efficiency of local visual processing and inferior parietal lobe played a key role in such cognitive visual-spatial function. Some researches shown decreases of gray matter reductions located in superior temporal sulsus, fronto-striatal and parietal network in autism [65,66]. But cerebral gray matter volume (especially in left-lateralized) increases were found for the individuals with autism [67-69], which is in accordance with the results in this study. It is important to note that our sample consisted of a typical population, which was regarded as neurotypical controls in autism researches. FI individuals with more efficient performance in EFT are more able to perceive an element independently from its context. According to our study, this stable personality characteristic may have its structural basis in the left IPL. Thus, increased rGMV in the left IPL in FI individuals might be associated with an excellent function of local visual processing. 

Secondly, higher ALFF in the brain areas belonging to the fronto-parietal network, including the left IPL and mPFC might primarily reflect the dis-embedding process during EFT. We found no apparent overlap between structural and functional findings. Haier et al. [38] indicated that structural change in one brain region did not necessarily result in functional change in the same location. No study has used anatomical and functional methods in combination in large normal sample to explore the neural basis of FDI cognitive styles. 

The fronto-parietal network, which includes elements of the dorsal attention network elements, mainly consists of IPL, IPS, ventromedial prefrontal cortex (vmPFC), precuneus, dorsal frontal, and midcingulate [70-72]. Evidence has suggested that the fronto-parietal network is responsible for attention, visuospatial working memory, and cognitive control [56,73]. Neuroimaging studies have demonstrated that the posterior parietal cortex area contributes as a key locus of storage of representation of visual information and is associated with feature identification in spatial stimuli processing [60,62,74-76]. Furthermore, many previous studies had indicated that the left temporo-parietal regions attend to local aspect of an object’s shape [12,13,15,77-79]. Regarding human visual system, objects within the visual scene are inclined to be identified and perceived as a whole [10,80], and this global perception tendency would dominate during EFT. FI individuals could perceive the local details less influenced by the global form. Therefore, higher ALFF in the left IPL might be related to superior feature identification of local processing for FI individuals. Moreover, we also found the significant positive correlation between ALFF in the mPFC and EFT score, which may also be comparable to previous neuropsychological studies that reported the involvement of the mPFC in cognitive styles. Previous functional imaging studies have indicated that the mPFC might be involved in high-level conflict monitoring, executive control, and decision making [81-86]. Normative perception has preferential global processing bias and better perform in EFT need inhibition of the global perceptual bias to reach more local processing [87,88]. Liu et al. [89] reported that greater activation in the superior frontal and medial frontal brain region in the line-counting task may reflect inhibition of automatic global processing of 3D configurations information, which may interfere with the concurrent local processing. Walter and Dassonville [14] also indicated that superior frontal gyrus might be involved in top-down control of attention required in search for the embedded figure. Furthermore, mPFC has been posited to reflect the suppression of the global perceptual bias during perception of local aspects of hierarchical stimuli [12,87,88]. In addition, The FI individuals could override the global perceptual bias to disembed a local component successfully. Thus, higher ALFF in mPFC for FI individuals was related to more effective at cognitive inhibition of field/global information, which may bring about detail-focused processing. 

Visual perceptual styles could affect an individual’s social cognition. A previous study has revealed that there existed significant relations between perceptual styles and social behavior [5]. Russell-Smith et al. [90] suggested that the superior EFT performance in autism may be strongly linked to the social deficits. “Autistic-like” traits, including the display of superior performance on the EFT, were continuously and normally distributed in the general population [91-93]. In our study, inferior parietal lobe (belonging to the tempoparietal junction, TPJ) and mPFC also served as vital regions of social cognition. Recent studies on autism have identified failing to deactivation at resting-state and found that the amount of functional abnormality in mPFC is positively correlated with that of social impairment [94,95]. However, evidence has suggested seeming different cognitive patterns was found between autistic traits in typical and ASD populations [96,97]. In the present study, a higher rest activation of the network was involved in the social cognition in FI individuals with high EFT scores. This result was consist with von dem Hagen et al.’s study [98]. They also found increased rest activation with greater autistic traits in the typical population. However, this study didn’t have sufficient evidence to test the relationship between local processing bias and social cognition directly. Future studies should address this issue to determine whether or not the differences in autism traits at the neuropsysiological level between the autism and normal populations.

In summary, the present study revealed the neural correlates of cognitive styles (FDI) by combining structural and functional MRI analyses in a large sample of healthy young adults. We found that increased rGMV in left IPL might be the structure basis of excellent local processing. Furthermore, functional results indicated that field-independent individuals might recruit a strong fronto-parietal network, relating to superior feature identification and cognitive inhibition. Finally, we attempted to point out that there might be different relationships between visual perceptual styles and discrepancy of individual’s social cognition in typical population and autism. The combination of structural and functional MRI methods possiblyprovided complementary information and advanced our understanding of the FDI cognitive styles. The correlation is arguably low for the research, which is probably due to the large sample size. The large sample analysis improves the validity of the neuroimaging research to some extent. In the future, we will choose clinical subjects with local brain lesion in the related regions in this research to further investigate the neural basis of FDI cognitive styles.
